# Multi-Layer Molecular Quantum-Dot Cellular Automata Multiplexing Structure with Physical Verification for Secure Quantum RAM

**DOI:** 10.3390/ijms26199480

**Published:** 2025-09-27

**Authors:** Jun-Cheol Jeon

**Affiliations:** Department of Convergence Science, Kongju National University, Gongju 32588, Republic of Korea; jcjeon@kongju.ac.kr

**Keywords:** molecular quantum-dot cellular automata, nanotechnology, multiplexer, physical verification, random access memory

## Abstract

Molecular quantum-dot cellular automata (QCA) are attracting much attention as an alternative that can improve the problems of digital circuit design technology represented by existing CMOS technology. In particular, they are well suited to the upcoming nanoquantum environment era with their small size, fast switching speed, and low power consumption. In this study, we propose a 5 × 5 × 1 ultra-slim vertical panel type multi-layer 2-to-1 multiplexer (Mux) using molecular QCA, departing from conventional multi-layer formats, and show its expansion to 4-to-1 Mux and application to vertical panel type D-latch and RAM cells. In addition, the polarization phenomenon of cells is physically proven using the potential energy, distance among electrons, and the relative positions of cells, and the secure RAM design takes noise elimination and polarization of the output signal into consideration. The circuits are simulated in terms of operation and performance using QCADesigner 2.0.3 and QCADesignerE, and the proposed multi-layer 2-to-1 Mux shows a significant improvement of at least 1473% and 277% in two representative standard design costs compared to the state-of-the-art multi-layer Muxes.

## 1. Introduction

Nanotechnology refers to technology that physically or chemically synthesizes, assembles, and controls nano-sized single atoms or molecules or measures and identifies their properties. Meanwhile, digital circuit design technology, represented by existing complementary metal–oxide–semiconductors (CMOSs), faces serious problems due to short channel effects, ultra-thin gate oxide, doping fluctuations, and expensive lithography at the nanoscale level [1]. Existing CMOS technology is reaching various physical limits that arise as hardware becomes smaller, and new alternatives are required due to problems such as large amounts of power leakage and the tunneling phenomenon. Accordingly, quantum-dot cellular automata (QCA) are attracting attention as a new alternative technology with small nanoscale space utilization, low power consumption, and high switching speed [2,3].

Recently, various logic circuits using QCA have been designed. In particular, due to the high interest in memory design [4,5], various designs for random access memory (RAM) are being attempted. RAM is an essential component in the digital circuit design of large storage devices and can be designed as a loop-based RAM cell or line-based RAM cell in QCA [6,7,8]. The loop-based RAM cell, which has a great advantage in latency, consists of a D-latch composed of a 2-to-1 multiplexer (Mux) and several gates that can be configured as a majority gate [9,10]. In other words, the 2-to-1 Mux has the greatest impact on the design of an efficient RAM cell. In addition, the Mux is a core component of computer systems and is an important core combinational circuit widely used in communication equipment such as routers, along with demultiplexers and ALU, which performs various arithmetic and logical operations [11,12,13,14,15,16,17,18].

The implementation of QCA has been attempted by various methods such as metal, semiconductor, magnetic, and molecular. The QCA cell was first implemented using metal quantum dots patterned on an insulating oxide [19] and was later implemented at room temperature using semiconductor quantum dots and silicon atom quantum dots [20,21]. Molecular quantum-dot cellular automata (QCA) is a low-power computing paradigm that can provide ultra-high device density and THz speed switching at room temperature [22,23,24]. The implementation of molecular QCA has been the most studied until recently, and most of the previous studies required chemical oxidation or reduction of molecules in an ionic state [25].

Meanwhile, despite the difficulty of future manufacturing and high design complexity, QCA-based multi-layer structures are being studied variously due to their advantages such as area utilization and fault tolerance [26,27,28,29]. Song et al. proposed a QCA-based low-cost RAM using a multi-layer structure [26], and Heikalabad et al. proposed a QCA full adder using three layers [27]. Chu et al. proposed a QCA-based BCD adder using a multi-layer structure [28], and Perri et al. proposed a QCA multi-bit full comparator using multi-layer crossover [29]. In addition, various QCA-based multiplexers using multi-layer structures have recently been developed [30,31,32,33,34,35,36], and excellent research that takes advantage of the multi-layer structure is actively underway.

The novelty and key contributions of the proposed research are as follows.

- Design of a 5 × 5 × 1 novel ultra-slim vertical panel type 2-to-1 Mux that is completely different from the existing molecular QCA-based multi-layer structure Mux.
- Physical verification through multiple lemmas and examples to prove the design principle of the proposed multi-layer 2-to-1 Mux.
- Design of a 4-to-1 Mux using three 2-to-1 Muxes to demonstrate the easy expansion of the proposed unit Mux.
- Design of an 8 × 13 × 1 vertical panel type RAM cell using the proposed unit Mux to demonstrate the modularity and easy application.
- The proposed structure demonstrates innovative excellence in all performance metrics and standard design costs compared to existing multi-layer Muxes.
- To design a safe quantum RAM circuit, the polarization of the output signal was maximized and noise was minimized.

This paper is structured as follows. Section 2 mentions the basic knowledge of QCA and existing multi-layer multiplexers, and Section 3 presents the proposal of the ultra-slim vertical panel QCA 2-to-1 multiplexer proposed in this study and physical proof of the cell interaction structure. Additionally, to demonstrate the scalability and applicability of the proposed structure, we provide an expanded 4-to-1 Mux and a vertical panel type RAM cell. In Section 4, the operation of the circuit is confirmed through simulation, performance is evaluated, and comparison and analysis are performed with existing studies. Finally, we conclude in Section 5.

## 2. Results

In this section, a multi-layer vertical panel type 2-to-1 multiplexer is proposed. It is extended to a 4-to-1 Mux and a loop-based RAM cell is also implemented. In addition, the normal operation and operating principle of the proposed multi-layer 2-to-1 multiplexer are physically demonstrated to understand it.

### 2.1. Proposed Vertical Panel Type 2-to-1 Multiplexer and Its Expansion and Application

**Figure 1:**
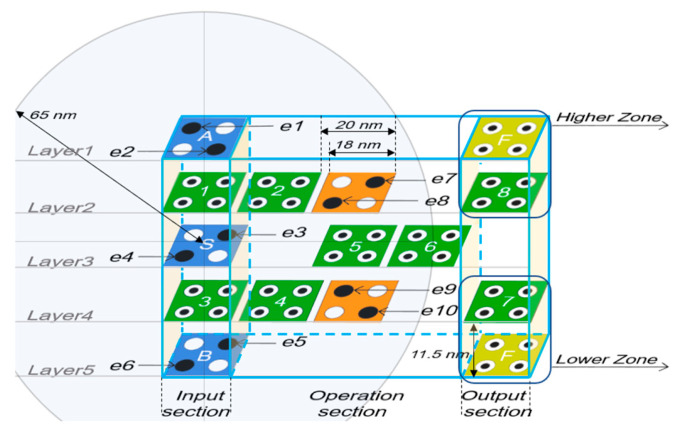
Proposed QCA-based 2-to-1 Mux with 5-layer structure, and the location of electrons in each layer and cell naming.

The multi-layered 2-to-1 Mux proposed in this study is shown in Figure 1. It consists of 5 layers. The values of *A* and *B* are input from the 1st and 5th layers, and one of the values of *A* and *B* is output according to the *S* value input from the 3rd layer. Looking at the proposed structure vertically, it consists of five columns, the first and fifth columns are called the input section and output section, respectively, and the three middle columns are called the operation section. The operation is performed with the input in the first clock phase, and cell 6 obtains the result of *F*′. At this time, by selecting a higher zone or lower zone in the output section, the resulting value, *F*, can be output to layer 1 or layer 5. Depending on the circuit being connected, the efficiency of circuit connection can be in-creased by using *F*′ of cell 7 or 8.

**Figure 2:**
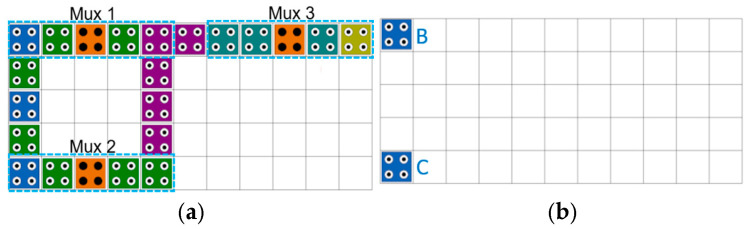

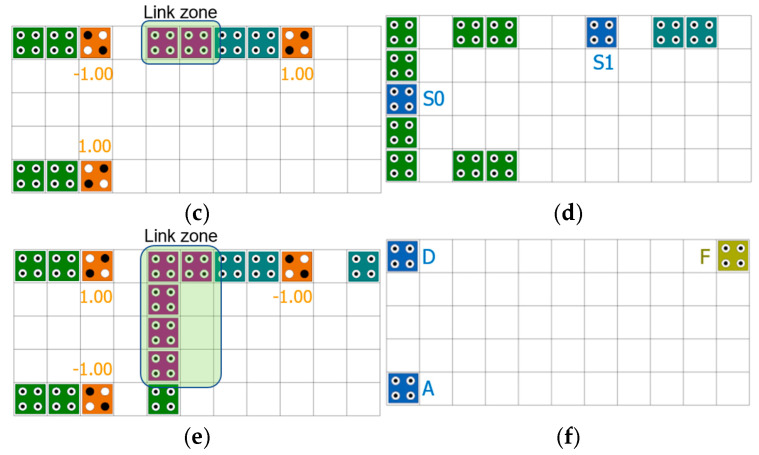
Proposed 4-to-1 Mux: (**a**) Top view; (**b**) Layer 1; (**c**) Layer 2; (**d**) Layer 3; (**e**) Layer 4; (**f**) Layer 5.

Figure 2 shows floor plans of the circuit that forms a 4-to-1 Mux using three 2-to-1 Muxes, and Figure 2a is a top view of the entire circuit. Mux1 has inputs *B* and *D*, Mux2 has inputs *A* and *C*, and selector S0 is located in the middle of the two Muxes. Mux1 and Mux2 select the higher zone and lower zone as output, respectively, and directly use the values from layer 2 and layer 4 as input to Mux3 using the link zone, as shown in Figure 2c,e. In other words, by directly connecting the positions of cells 8 and 7 in Figure 1 to the inputs of cells 1 and 3 of Mux3, two inverter operations are eliminated each, thereby increasing computational efficiency. Mux3 uses the second selector S1, selects the lower zone, and outputs F to layer 5.

Table 1 shows the truth table of the 4-to-1 Mux, and the input values, *A*, *B*, *C*, and *D*, are output according to the value of the selection pair of *S*0 and *S*1.

**Figure 3:**
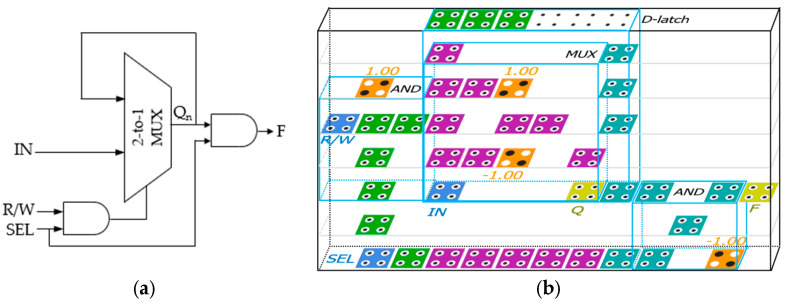
A loop-based RAM cell using the proposed 2-to-1 Mux Module: (**a**) a logic diagram; (**b**) QCA layout.

The proposed structure is an ultra-slim vertical panel type 5 × 5 × 1 modular circuit with a thickness of 18 nm and is designed as a selectable structure with the option to choose the output unit depending on the surrounding environment. Figure 3b shows an ultra-slim vertical panel RAM cell of 8 × 13 × 1 using the proposed 2-to-1 Mux structure, and Table 2 shows the truth table of RAM cell with three inputs shown in Figure 3a. The proposed RAM cell is highly stable and modular and can be easily expanded to n × m RAM structures. A RAM cell operates only when SEL has a value of 1, otherwise it always outputs 0 regardless of the previous output value. When the value of SEL is 1 and R/W is 0, a read function is performed and the output value is the previous output value regardless of the IN value. When R/W is 1, a write function is performed and the value of IN is output regardless of the previous value.

### 2.2. Physical Verification of 2-to-1 Multiplexer

The proposed 2-to-1 Mux is designed with a multi-layer structure based on cell interaction, so it is hard to logically prove the results. Therefore, in this section, we physically prove the operation of the proposed circuit using the potential energy generated by the Coulomb repulsion among electrons. For physical verification, several assumptions are required.

**Assumption** **1.** *The following five assumptions are made regarding parameters and environment for physical verification.*

*1.*  *The size of each cell is 18 nm × 18 nm.*
*2.*  *Each cell has a 2 nm distance from neighboring cells in the same layer.*
*3.*  *The distance between each layer is 11.5 nm.*
*4.*  *The radius that can affect electrons is 65 nm.*
*5.*  *Four quantum dots are located at each corner of a square cell.*

In the proposed Mux, when the input values of *A*, *B*, and *S* cells and the values of fixed cells are determined as shown in Figure 1, each electron is named e1 to e10 for physical verification. Equation (1) represents the potential energy between two electrons, *q*_1_ and *q*_2_. *U* is potential energy, *k* is Coulomb’s constant of 8.9875 × 10^9^ Nm^2^/C^2^, *q*_1_ and *q*_2_ represent the amount of electric charges of 1.6 × 10^−19^ C each, and *r* represents the distance between two electrons in meters (m). By entering values for *k* and *q*, *kq*_1_*q*_2_ can be expressed as Equation (2), and all factors have a value set by *C*, a constant value representing the Coulomb repulsion. Additionally, the formula for calculating the sum of potential energy, *U_T_*, appears in Equation (3) [37,38].

(1) $$U=\frac{{kq}_{1}q_{2}}{r^{2}}$$

(2) $$C=kq_{1}q_{2}=8.9875\times 10^{9}\times \left(1.6^{2}\right)\times 10^{-38}=23.04\times 10^{-29}$$

(3) $$U_{T}=\sum_{i=1}^{n}U_{i}$$

**Lemma** **1.** *The polarization of a cell is determined by the smaller sum of potential energies of electrons within a radius that can be affected by the cell at a specific time.*

**Proof.**  Low potential energy with electrons means low repulsion from affected electrons, so it is determined by the value of the corresponding polarization. □

**Figure 4:**
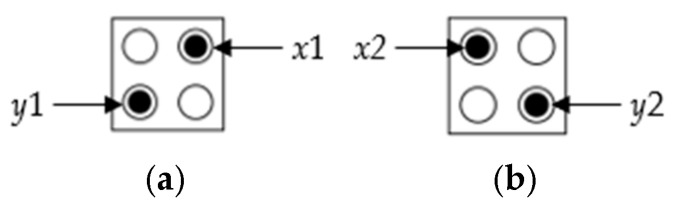
Assumptions of electron positions: (**a**) the positions of *x*1 and *y*1 when *P* = +1, (**b**) the positions of *x*2 and *y*2 when *p* = −1.

Let us find the polarization of cell 2 in Figure 1. The potential energy of electron *x*1 can be obtained as the sum of the potential energies of all electrons from e1 to e10 entering the radius of effect. As shown in Figure 4a, when the value of cell 2 is *p* = +1, if the electrons are *x*1 and *y*1, the sum of each potential energy can be obtained as in Examples 1 and 2 below.

**Example** **1.** *Let us find the sum of the potential energies of electrons e1 to e10 and x1 of cell 2.*

$U_{e1x1}=\frac{23.04\times 10^{-29}}{15.76\times 10^{-16}}=1.46\times 10^{-13}$



$U_{e3x1}=\frac{23.04\times 10^{-29}}{5.32\times 10^{-16}}=4.33\times 10^{-13}$



$U_{e5x1}=\frac{23.04\times 10^{-29}}{15.90\times 10^{-16}}=1.45\times 10^{-13}$



$U_{e7x1}=\frac{23.04\times 10^{-29}}{4\times 10^{-16}}=5.75\times 10^{-13}$



$U_{e9x1}=\frac{23.04\times 10^{-29}}{5.33\times 10^{-16}}=4.32\times 10^{-13}$



$U_{e2x1}=\frac{23.04\times 10^{-29}}{8.56\times 10^{-16}}=2.69\times 10^{-13}$



$U_{e4x1}=\frac{23.04\times 10^{-29}}{19.00\times 10^{-16}}=1.21\times 10^{-13}$



$U_{e6x1}=\frac{23.04\times 10^{-29}}{29.58\times 10^{-16}}=0.78\times 10^{-13}$



$U_{e8x1}=\frac{23.04\times 10^{-29}}{3.28\times 10^{-16}}=7.02\times 10^{-13}$



$U_{e10x1}=\frac{23.04\times 10^{-29}}{12.53\times 10^{-16}}=1.84\times 10^{-13}$



The sum of the potential energies of each electron and *x*1 is given in Equation (4).

(4) $$U_{Tx1}=\sum_{i=1}^{10}U_{i}=3.09\times 10^{-12}$$

**Example** **2.** *Let us find the sum of the potential energies of electrons e1 to e10 and y1 of cell 2.*

$U_{e1y1}=\frac{23.04\times 10^{-29}}{8.56\times 10^{-16}}=2.69\times 10^{-13}$



$U_{e3y1}=\frac{23.04\times 10^{-29}}{4.64\times 10^{-16}}=4.97\times 10^{-13}$



$U_{e5y1}=\frac{23.04\times 10^{-29}}{15.18\times 10^{-16}}=1.58\times 10^{-13}$



$U_{e7y1}=\frac{23.04\times 10^{-29}}{17.68\times 10^{-16}}=1.30\times 10^{-13}$



$U_{e9y1}=\frac{23.04\times 10^{-29}}{1.57\times 10^{-16}}=14.67\times 10^{-13}$



$U_{e2y1}=\frac{23.04\times 10^{-29}}{3.28\times 10^{-16}}=7.02\times 10^{-13}$



$U_{e4y1}=\frac{23.04\times 10^{-29}}{5.32\times 10^{-16}}=4.33\times 10^{-16}$



$U_{e6y1}=\frac{23.04\times 10^{-29}}{7.49\times 10^{-16}}=3.08\times 10^{-16}$



$U_{e8y1}=\frac{23.04\times 10^{-29}}{4\times 10^{-16}}=5.75\times 10^{-16}$



$U_{e10y1}=\frac{23.04\times 10^{-29}}{19.73\times 10^{-9}}=1.17\times 10^{-16}$



The sum of the potential energies of each electron and *y*1 is given in Equation (5).

(5) $$U_{Ty1}=\sum_{i=1}^{10}U_{i}=4.66\times 10^{-12}$$

Therefore, when cell 2 is *p* = +1, the sum of potential energy can be obtained by Equation (6).

(6) $$U_{T1}=U_{Tx1}+U_{Ty1}=3.09\times 10^{-12}+4.66\times 10^{-12}=7.75\times 10^{-12}$$

By the same method, when the value of cell 2 is *p* = −1 as shown in Figure 4b, if the electrons are *x*2 and *y*2, the sum of each potential energy can be obtained in Equations (7) and (8).

(7) $$U_{Tx2}=\sum_{i=1}^{10}U_{i}=3.49\times 10^{-12}$$

(8) $$U_{Ty2}=\sum_{i=1}^{10}U_{i}=8.53\times 10^{-12}$$

Therefore, when cell 2 is *p* = −1, the sum of potential energy can be obtained by Equation (9).

(9) $$U_{T2}=U_{Tx2}+U_{Ty2}=3.49\times 10^{-12}+8.53\times 10^{-12}=12.02\times 10^{-12}$$

As shown in Equations (6) and (9), the value of $U_{T1}$ was significantly low, resulting in the conclusion that it is correct for cell 2 to be located at *x*1 and *y*1. Therefore, in the situation shown in Figure 1, the value of cell 2 has *p* = +1. This result shows that the value of $U_{e8y2}$ is much higher than other average values, which produces a severe Coulomb repulsion because the distance to the electrons in the neighboring fixed cell is too close.

## 3. Discussion

All circuits were simulated using QCADesigner 2.0.3 [39], a bistable approximation simulation engine was used, and the related parameters are summarized as follows. Cell size: 18 nm, dot diameter: 5 nm, cell separation: 2 nm, layer separation: 11.5 nm, clock high: 9.8 × 10^−22^ J, clock low: 3.8 × 10^−23^ J, clock shift: 0, clock amplitude factor: 2.0, relative permittivity: 12.9, number of samples: 12,800, maximum iterations per sample: 100, convergence tolerance: 1.0 × 10^−3^, radius of effect: 65 nm, and randomized simulation order.

**Figure 5:**
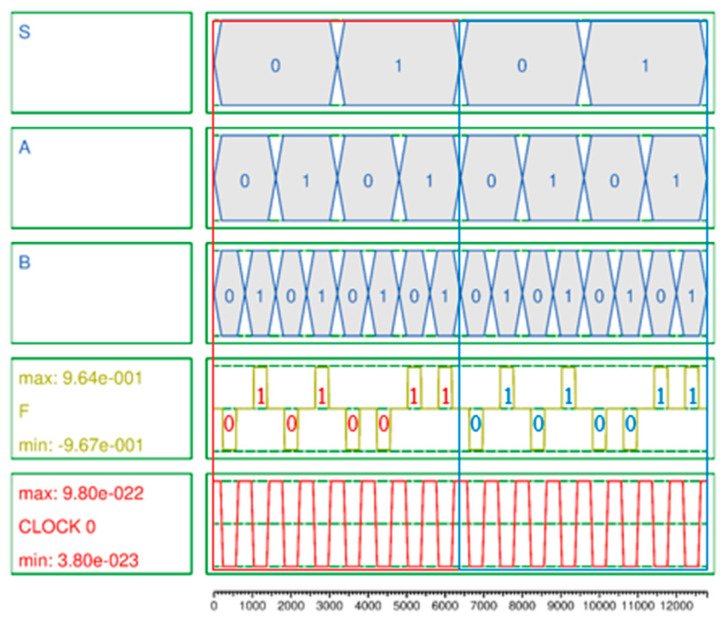
Simulation result of proposed multi-layer 2-to-1 Mux.

**Figure 6:**
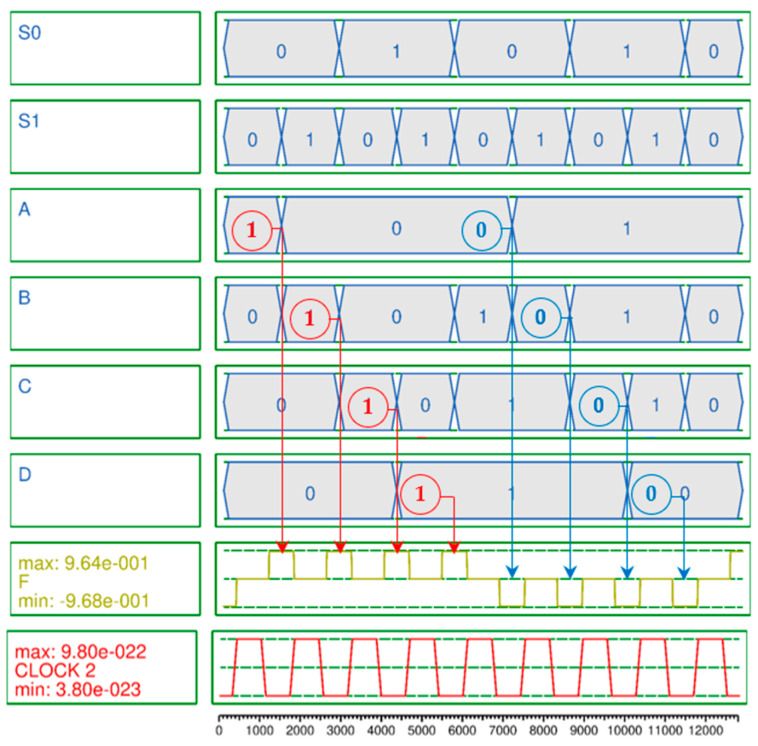
Simulation result of proposed QCA-based 4-to-1 Mux.

The simulation result of the 2-to-1 Mux shown in Figure 5 is accurately output according to Equation (13) with eight outputs in one cycle at clock 0, the first clock phase. Meanwhile, to effectively test the proposed 4-to-1 Mux, a vector table is created as the input of the circuit and simulation is performed. The simulation consists of two cycles. In the first and second cycles, the vector tables are designed so that the input values are all 1 and 0, respectively. Otherwise, it is confirmed that it is malfunctioning. Figure 6 is the simulation result of the proposed 4-to-1 Mux, and it was confirmed that the normal result was output using only three clock phases in clock 2 according to Table 3.

**Figure 7:**
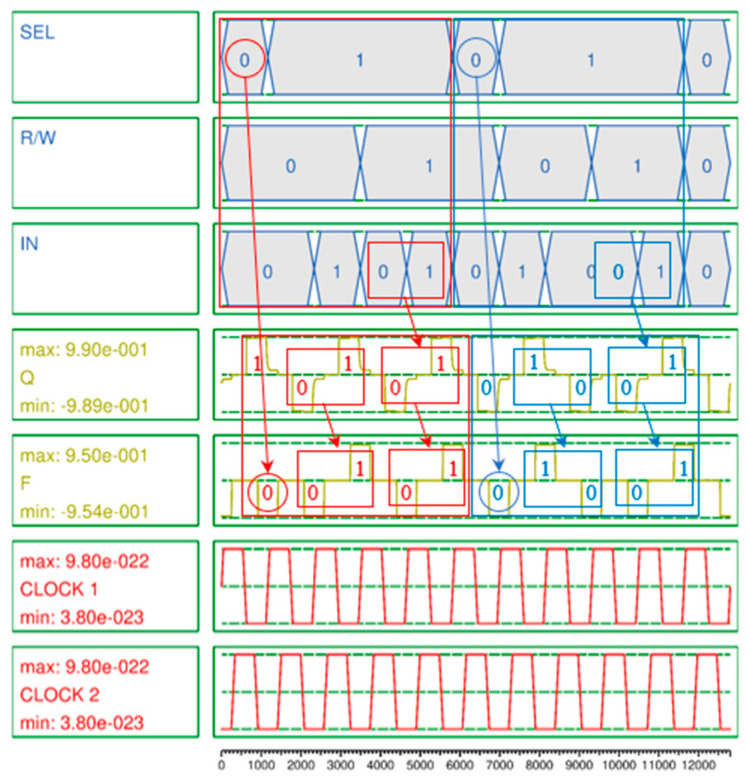
Simulation result of proposed QCA-based RAM cell.

Figure 7 is the simulation result for a RAM cell using the proposed QCA-based 2-to-1 Mux. This simulation result shows that if SEL = 1 and R/W = 0, *F* has the value of Q, and if SEL = 1 and R/W = 1, *F* has the value of IN, and otherwise, *F* = 0, as shown in Table 1.

A secure circuit is closely related to the polarization strength and noise of the output signal. This is because the abilities to continuously maintain or update the value stored in the QCA cell and preserve that value during circuit expansion and connection, and to ensure that the value is not distorted by weak polarization and noise, are crucial aspects of RAM that must maintain values for long periods of time. Typically, a stable polarization has a strength of about 9.5e−001. The average output polarizations (AOPs) of the proposed 2-to-1 Mux and 4-to-1 Mux are 9.655e−001 and 9.66e−001 as shown in Figure 5 and Figure 6, respectively, which exceeds the average AOP. In particular, the AOP of the D-latch is 9.895e−001, which provides a very high output polarization for the RAM output, so that the RAM output, F, can safely maintain its value without any noise or distortion and store or update its value as shown in Figure 7.

Meanwhile, in order to compare the performance of the circuits, the most basic performance metrics, cell count, area (nm^2^), delay (clock cycle), and energy dissipation (10^−2^ eV), and two representative design costs, $Cost_{AD}$ and $Cost_{ED}$, shown in Equations (10) and (11) are compared with existing multi-layer structures.

(10) $$Cost_{AD}=A\times D^{2}$$

where A and D refer to the area and the delay of a circuit, respectively. Equation (11) is a standard design cost measurement method including energy dissipation and delay [38,40].

(11) $$Cost_{ED}=E^{2}\times D^{2}$$

where E and D refer to the energy dissipation and the delay of a circuit, respectively. Equation (12) is applied to the area, delay is expressed in units of one clock cycle with four clock phases, and energy dissipation is obtained using QCADesignerE [41].

(12) $${Area}_{multi-layer}={Area}_{reported}\times m$$

where m is the number of layers on a multi-layer structure to reflect the higher area cost of a multi-layer design over a coplanar structure [28,38,40]

**Figure 8:**
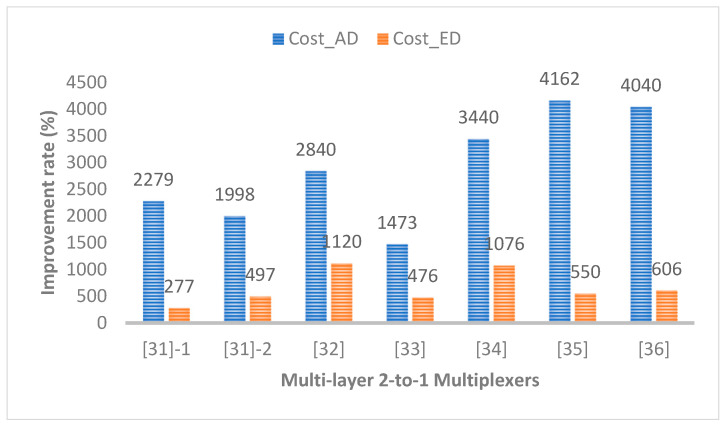
Improvement rates of two representative design costs compared to typical multi-layer 2-to-1 Muxes.

Table 4 shows that the proposed structure has greatly superior results compared to existing multi-layer 2-to-1 Mux structures. Although the proposed circuit is not the best in terms of energy dissipation, it has a trade-off with delay and the associated design cost must be checked. As shown in Figure 8, the proposed structure showed tremendous improvements of at least 1473% and 277% compared to the existing circuit in two representative standard design costs, $Cost_{AD}$ and $Cost_{ED}$.

## 4. Materials and Methods

### 4.1. Basic Knowledge of QCA

**Figure 9:**
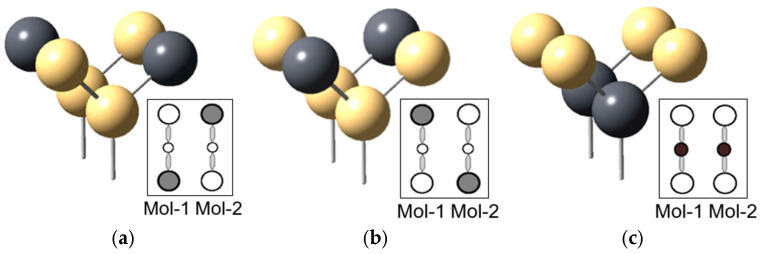
Polarizations of Molecular QCA with six quantum dots [31]: (**a**) *p* = +1, (**b**) *p* = −1, (**c**) *p* = null.

In the molecular QCA structure, the QCA cell consists of six quantum dots and two electrodes as shown in Figure 9, and one electron stays on each electrode to encode the value of binary information according to the charge distribution. Figure 9a,b show the state where the electrons are located in the diagonal direction and the polarization of the cell is *p* = −1 and *p* = +1, respectively, and Figure 9c shows the state where the electrons are located in the middle of the electrodes and maintain the null state [42,43].

**Figure 10:**
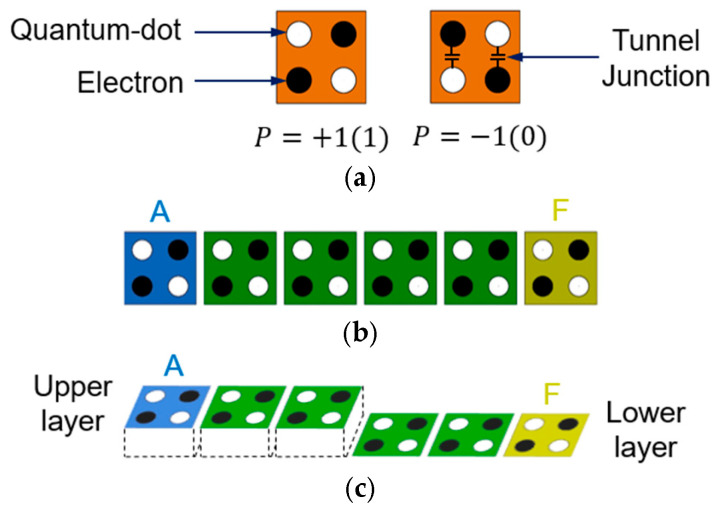
QCA cells: (**a**) regular cells with polarization “+1” and “−1”; (**b**) wiring on single layer; (**c**) wiring between different layers.

The most recent and widely used QCA modeling is as shown in Figure 10. A QCA cell has four quantum dots at each corner of a square, as shown in Figure 10a, and is composed of two moving electrons. Between quantum dots, there is a tunnel through which electrons can move. Electrons always exist on the diagonal due to Coulomb repulsion, which pushes them away from each other, and they have two polarizations: +1 and −1. This corresponds to 1 and 0 in binary operations. Figure 10b shows the QCA wire, which can be easily constructed by placing several cells in a row. Figure 10c shows the wiring between different layers [2,3].

**Figure 11:**
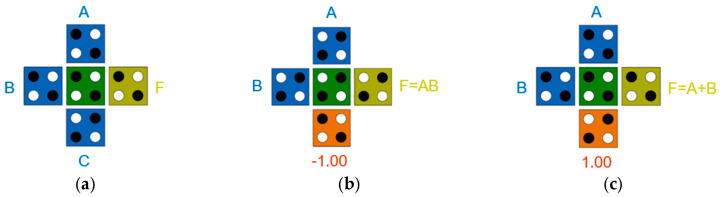

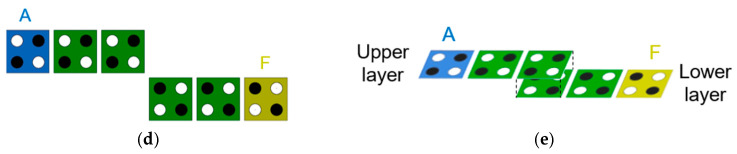
QCA gates: (**a**) majority gate with inputs (A, B, C) and output (F); (**b**) AND gate with fixed cell (*p* = −1); (**c**) OR gate with fixed cell (*p* = +1); (**d**) a simple inverter on the same plane; (**e**) a simple inverter between different layers.

Figure 11 shows the basic logic gates required for QCA circuit design. Figure 11a shows a majority vote gate with three inputs (A, B, C) and one output F. At this time, by fixing one input to −1 or +1, the product or sum of the two inputs A and B can be obtained as shown in Figure 11b,c. Figure 11d,e show a simple inverter in a single plane and between different layers [44].

**Figure 12:**
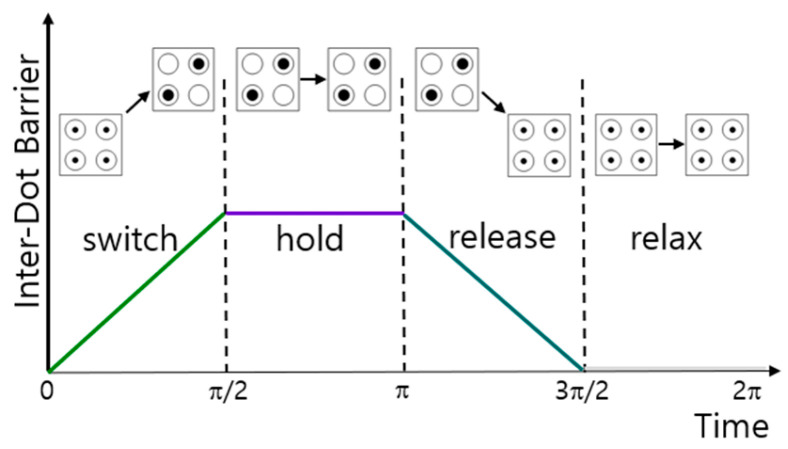
Changes of Inter-dot barrier between quantum dots and QCA states over time.

Figure 12 shows four QCA clock states depending on the change in time and barrier between quantum dots [45]. A QCA cell operates in the following four states. The state in which the barrier between quantum dots gradually increases is called a switch, and the state in which the barrier is sufficiently high enough that electrons cannot move is called a hold. At this time, the polarization of the cell is determined. The state in which the barrier is gradually lowered is called release, and the state in which the barrier is sufficiently lowered so that electrons can move actively is called relax.

### 4.2. QCA-Based Multi-Layer Multiplexer

A 2-to-1 Mux is one that receives two values, *A* and *B*, as input, determines one of the input values using a selector *S*, and sends it to the output line. Equation (13) represents the logical expression of Mux as majority gate function (MG) with three inputs: *A*, *B*, and *S* [46].

(13) $$F=AS^{\prime }+BS=MG\left(MG\left(A,S^{\prime },-1\right),\;MG\left(B,S,-1\right),1\right)$$

Mux is a logic circuit used as a component of various combinational and sequential circuits, and various types of research have been conducted using QCA. Initially, it started with majority gate-based circuits [46,47] and progressed to XOR gate-based circuits [47,48] and cell interaction-based circuits [49,50]. Recently, research has expanded to include circuits that utilize the characteristics of multi-layer structures.

In 2008, Hashemi et al. proposed a novel multi-layer multiplexer that connects selectors with multi-layer crossovers [30]. In 2015, Sen et al. proposed two multi-layer multiplexers with three and four layers while minimizing the area [31]. After that, in 2019, Mosleh proposed a three-layer multiplexer using the MV32 majority gate with two outputs [32]. In 2020, Singh et al. designed a Mux using a multi-layered XOR gate [33] and proposed a RAM cell using a multi-layered Mux [34]. In the same year, Seo et al. proposed a loop-based RAM cell using a multi-layer Mux [35], and in 2023, Jain et al. proposed a Mux using a rotated majority gate and a multi-layer crossover and a single-bit RAM cell was proposed [36]. Research on the multi-layered 2-to-1 Mux was difficult due to the complexity of the design and the uniqueness of the structure, but various studies have been presented using new creative ideas. Research on existing multi-layer Mux structures was designed for efficient RAM cell design and was often used as a crossover to connect multiple gates. The truth table of the 2-to-1 Mux is shown in Table 3.

## 5. Conclusions

The proposed study designed a 2-to-1 QCA multiplexer with a multi-layer structure and demonstrated the internal operation by cell interaction using physical proof, and the performances and standard design costs showed significant improvements compared to existing studies. In particular, the proposed structure is designed as an ultra-slim vertical panel type and has excellent expandability and applicability, which was confirmed through the design of a 4-to-1 Mux and RAM cell. The proposed structure is a completely new and challenging format that deviates from the existing multi-layer design format and can serve as a guideline for future three-dimensional QCA circuit design. In addition, due to its high modularity and low design cost, it can be a major component in many circuit designs using a multi-layer Mux. With the rapid development of 3D stacked memories such as HBM in semiconductors, the feasibility of multi-layer structures in QCA is increasing, and QCA technology will further develop through challenging research.

## Figures and Tables

**Table 1:** Truth table of 4-to-1 Mux (’X’ means ’Don’t care’).

S0	S1	A	B	C	D	F
0	0	0	X	X	X	0
0	0	1	X	X	X	1
0	1	X	0	X	X	0
0	1	X	1	X	X	1
1	0	X	X	0	X	0
1	0	X	X	1	X	1
1	1	X	X	X	0	0
1	1	X	X	X	1	1

**Table 2:** Truth table of RAM cell.

SEL	R/W	IN	Q_n-1_	Q_n_	F
0	X	X	X	Q_n-1_	0
1	0	X	0	0 (Q_n-1_)	0 (Q_n-1_)
1	0	X	1	1 (Q_n-1_)	1 (Q_n-1_)
1	1	0	X	0	0
1	1	1	X	1	1

**Table 3:** Truth table of 2-to-1 Mux.

S	A	B	F
0	0	0	0
0	0	1	1
0	1	0	0
0	1	1	1
1	0	0	0
1	0	1	0
1	1	0	1
1	1	1	1

**Table 4:** Performance comparison of Multi-layer 2-to-1 Mux.

Circuit	Cell		Area		Delay		Ener. Dissi.		Cost_AD_		Cost_ED_	
	no.	ratio	nm^2^	ratio	clocks	ratio	10^−2^ eV	ratio	AD^2^	ratio	E^2^D^2^	ratio
[30]	36	2.77	122,292	13.87	1.00	4.00	1.85	1.32	122,292	221.84	3.42	27.94
[31]-1	23	1.77	52,452	5.95	0.50	2.00	1.36	0.97	13,113	23.79	0.46	3.77
[31]-2	22	1.69	46,256	5.24	0.50	2.00	1.71	1.22	11,564	20.98	0.73	5.97
[32]	21	1.62	28,812	3.27	0.75	3.00	1.63	1.16	16,207	29.40	1.49	12.20
[33]	21	1.62	34,692	3.93	0.50	2.00	1.68	1.20	8673	15.73	0.71	5.76
[34]	24	1.85	34,692	3.93	0.75	3.00	1.60	1.14	19,514	35.40	1.44	11.76
[35]	25	1.92	41,772	4.74	0.75	3.00	1.19	0.85	23,497	42.62	0.80	6.50
[36]	20	1.54	40,572	4.60	0.75	3.00	1.24	0.89	22,822	41.40	0.86	7.06
Ours	13	1.00	8820	1.00	0.25	1.00	1.40	1.00	551	1.00	0.12	1.00

## Data Availability

Data are contained within the article.
